# Management of Type 2 Diabetes Mellitus by Traditional Medicine Practitioners in Kenya- Key Informant Interviews

**DOI:** 10.11604/pamj.2015.22.90.6485

**Published:** 2015-10-01

**Authors:** Irene Njeri Chege, Faith Apolot Okalebo, Anastasia Nkatha Guantai, Simon Karanja, Solomon Derese

**Affiliations:** 1 College of Health Sciences, Jomo Kenyatta University of Agriculture and Technology, Nairobi, Kenya; 2 School of Pharmacy, College of Health Sciences, University of Nairobi, Nairobi, Kenya; 3 Department of Chemistry, University of Nairobi, Nairobi, Kenya

**Keywords:** Herbalists, diabetes mellitus, traditional medicine

## Abstract

**Introduction:**

Worldwide, plant based medicines are increasing in popularity due to perceptions of safety and efficacy. Herbalists in Kenya are widely consulted for the management of many diseases including Type 2 Diabetes Mellitus (T2DM). This study investigated the level of knowledge of the herbalists in management of T2DM.

**Methods:**

Purposive sampling was used to identify 4 herbalists working in the urban areas who actively manage T2DM. Key informant interviews were used to gather data about the management of T2DM. It was analyzed using a content thematic approach.

**Results:**

Diverse management methods which included both pharmacological and non- pharmacological were noted. Glycemic control was assessed with the help of a glucometer. In addition, presenting signs and symptoms were key in diagnosing T2DM. The herbalists used various herbs, minerals and animals as medicinal sources. The drugs were dispensed as decoctions with excipients being added appropriately. Adverse effects were recorded. The herbalists acknowledged that patients use both herbal and allopathic medicine together. A level of record keeping was observed but patient follow-up was poor. The cost of the herbal drugs was perceived to be excessive.

**Conclusion:**

Some similarities exist in the management of T2DM between allopathic and traditional medicine practitioners. Training of herbalists is required to improve the quality of care given to patients.

## Introduction

Traditional medicine practitioners (TMPs) or herbalists are lay persons or educated persons who claim to have the ability to cure disease conditions or provide symptomatic management using non-conventional approaches [1]. It has been reported that 75 - 90% of the populations in developing countries rely on traditional remedies for treatment of various diseases [2]. Patients procure their services because they perceive herbal treatments as affordable, easily available and acceptable compared to allopathic medicines [3, 4]. TMPs are also easily accessible and offer more personalized care compared with conventional doctors [5]. Poor outcomes with allopathic drugs, especially for chronic conditions also makes TMPs popular alternatives [6, 7]. One of the major chronic ailments widely managed by using TMPs is Type 2 Diabetes Mellitus (T2DM). Diabetes Mellitus (DM) is an endocrine disorder classified as either insulin dependent (T1DM) or non-insulin dependent (T2DM). Normally, insulin, a hormone produced by the β-cells of the pancreas, lowers blood glucose levels and its absence or insufficient production results in diabetes. T2DM is a syndrome characterized by hyperglycemia, insulin resistance and hyperlipidemia. Eventually, an untreated patient suffers from microvasular and macrovascular damage to the body that leads to diabetic complications which include retinopathy, nephropathy, neuropathy, diabetic foot, amputations, myocardial infarction and stroke [8]. In Sub-Saharan Africa, T2DM accounts for over 90% of diabetes cases with the population prevalence in urban areas of Kenya estimated at 12% [8] and 2.2% in rural areas [9]. In managing T2DM, patients seek conventional or traditional health care or a combination of both systems. This paper seeks to describe the management of T2DM by TMPs in Kenya and also assess their knowledge about the disease.

## Methods

A descriptive, cross-sectional study was conducted at the School of Pharmacy, University of Nairobi (UoN) and clinics in Nairobi County and its environs. Four TMPs swith at least five years experience in managing T2DM, were selected using purposive sampling and interviewed using interviews and guides. These TMPs had long standing collaborations in herbal medicine with the UoN. Purposive sampling was used since this research aims to develop an interpretative framework of use of herbal medicines in T2DM [10]. Moreover, purposeful sampling is the most appropriate method for documenting secretive events and rich information sources [11, 12]. According to the principles of qualitative research methodology, a sample size of two is adequate for an in-depth interview [12]. An interview guide was designed and used to collect information from TMPs on treatment and management of T2DM. Consent to participate in the main study was obtained 24 hours before the interview. Themes were identified and content thematic approach was used to analyze obtained data. The main themes were knowledge acquisition, self-beliefs about T2DM, source of herbs and patients, diagnosis and monitoring of patients and maintenance of patient records. Relevant verbatim quotations in the thematic areas were used to present the study findings. Ethical permission was acquired from the UoN/KNH Ethics and Research committee at Kenyatta National Hospital (KNH) (reference: KNH-ERC/A/306). The basic principles defined in Guidance for Good Clinical Practice and Declaration of Helsinki were followed [13]. Pseudo names were used in the transcripts to maintain confidentiality and the herbalists and their clinics were only identified by study numbers.

## Results

### Traditional medical practitioners’ knowledge about type 2 diabetes mellitus

All TMPs defined diabetes mellitus as the “sugar disease” in various languages. Three TMPs had gained informal training about T2DM; one had received formal training on T2DM. Two TMPs cited family as a source of their knowledge and skills. All four had attended various seminars on diabetes organized for herbalists by various educational institutions. They had also interacted with scientists at University of Nairobi. Experience, observation, research and experimentation over several years had honed their skills on diabetes management. The TMPs gave varied causes of the disease. They ranged from failure of tissues to respond to insulin (1), worm infestation leading to damage of the liver and pancreas (1), poor general health (2), stress (3), poor lifestyle (4), poor diet (4), lack of certain minerals (1) and family connection (2). Herbalist 4 stated that; *Worms can cause T2DM. They weaken the liver and the pancreas. It is also due to failure of tissue to respond to insulin and general poor health. (TMP 4)*

Herbalist 2 stated; *Lack of minerals is an important cause of T2DM. (TMP 2)*

### Sources of patients and patient referrals

Two sources of patients were identified; referrals and walk-ins. All TMPs stated that they had “many” patients. All TMPS relied on the use of referrals from patients that they had previously treated successfully. Two TMPs had well-established clinics whose services were advertised at the entrances. All TMPs affirmed that patient selection was practiced since very sick patients were referred for conventional treatments.

Herbalist 1 states that; “I have patients in many parts of Kenya, Tanzania, Uganda and Congo. I do not however accept very sick patients and prefer to refer them for conventional treatment. I do not take risks.” (TMP 1)

### Methods used to diagnose type 2 diabetes mellitus by traditional medicine practitioners

The methods used by these TMPs to diagnose T2DM are listed in Table 1. All four herbalists had access to glucometers. TMP1 had been robbed of his glucometer but insisted that patients should go to hospital for monitoring of fasting and random blood glucose levels. TMP2 and 4 owned their own while TMP3 relied on hospital results. Reference ranges were those stated in the glucometer inserts or in the hospital reports. Herbalists who owned their own glucometers were concerned about the price and availability of strips. Laboratories tests were requested for if the patients could afford them. One herbalist said; *“I do not use the glucometer if the patient knows the problem. I only use it if the patient does not know the problem." (TMP2)*

**Table 1:** Diagnostic methods used by TMPs

Methods of diagnosing T2DM	Glucometer (to measure FBS and RBS)	Hospital tests ^à^	Patient reported signs and symptoms	Previous diagnosis
**Herbalist 1**	P	P	P	P
**Herbalist 2**	P	O	P	P
**Herbalist 3**	P	O	P	P
**Herbalist 4**	P	P	P	O

à Hospital tests included kidney, liver and pancreatic tests function tests. FBS- Fasting Blood Glucose. RBS- Random Blood Glucose.

Herbalist 4 commented; *“The only objective criteria is pre and post glucose levels.”(TMP 4)*

Patient reported signs and symptoms that were of interest to the herbalist are listed in Table 2. All herbalists relied on the presence of at least 4 clinical signs with the exception of one herbalist who only stated one sign; sweating. This was the only herbalist who stated sweating was a clinical sign of T2DM. Three signs that were not accepted in conventional medicine were fever, sweating and headache. No patient examinations were observed in this study. Conversations between the herbalist and their patients were used to diagnose and monitor reported signs and symptoms that were then used to constitute a diagnosis.

**Table 2:** Patient reported signs and symptoms used as diagnostic parameters by various herbalists

Signs and Symptoms		Herbalist 1	Herbalist 2	Herbalist 3	Herbalist 4
**Early presenting signs**	Excessive urination	P	P	O	P
	Increased thirst	P	P	O	P
	Unusual hunger	P	O	O	P
	Weight changes	P	O	O	P
**Known complications**	Lack of sleep	P	O	O	O
	Male sexual weakness	P	O	O	O
	Poor appetite	P	O	O	O
	Poor vision	P	P	O	O
**Signs that are not typically associated with DM**	Headache	O	P	O	O
	Sweating	O	O	P	O
	Fever	O	P	O	O

### Maintenance of patient records

TMP3 had no patient records. TMP2 and 4 had recording systems consisting of books and cards which were examined by the researcher. They included information such as name, age, contacts, signs and symptoms, duration of diseases, diagnosis, drug dispensed and costs. Herbalist 1 recorded patient details in exercise books which were then returned to them. *“Patients keep their own records in the form of exercise books which I fill during patient visits. They are easy to access.” (TMP 1)*

None of the TMPs could state with accuracy the number of patients they had treated in the last one year including patients with T2DM.

### Treatment approaches for management of type 2 diabetes mellitus

Four treatment approaches were identified; plants, animals, minerals and lifestyle advice. All the four herbalists recommend their herbal formulations as the treatments of choice for T2DM. The number of herbs used by the four herbalists range from 1 to 14 individual herbs in their formulations. TMP1 also recommended animal products as part of the treatment. He favored specific animals body parts prepared in intricate ways to accompany his herbal medicines. These were also considered trade secrets. Herbalist 2 recommended the use of minerals such as magnesium chloride as adjunctive treatment. *“I use a combination of herbs that are synergistic. No single herb can be singled out. This combination is a trade secret.” (TMP 1)* *“I use a combination of herbs. This is a trade secret.” (TMP 2)*

TMP3 and 4 were willing to divulge their plant sources with the latter saying that his products were clearly labeled. Herbalist four noted that: *“Diabetes is curable depending on what has caused it. I treat the root cause and not the symptoms. Not all patients are treated with hypoglycemic herbs. Some patients have poor general health. The first treatment approach is to manage the poor general health by administering detoxifying herbal agents. Enemas and cathartics may be given. Some patients respond well by administering deworming agents. For patients who do not respond, a mono-component agent is used first.” (TMP 4)*

Herbalist one stated that sweet tasting and pleasant smelling plants were best to treat T2DM. Most of his herbs were observed to have these characteristics. Herbalist 2 stated that bitter tasting herbs with anti-malarial like qualities were most likely candidates for treating T2DM.

*“Any plant that treats malaria is also a treatment for diabetes.” (TMP 2)*

### Dispensing of drugs, dosage and duration of treatment

Three herbalists dispensed their medicines as powders while TMP 2 dispensed his as suspensions. The dosages given by TMPs were not standardized as the herbalists gave instructions such as, *“take one cup three times daily”*, or *“mix one cup in four cups of boiling water and simmer for 30 minutes”*. Instructions given by a second TMP were: *“Mix the herb in the ratio of 1:1. Boil the one teaspoon in a cup of water for 5-10 minutes. Take half a cup twice daily.” (TMP 3)*

All TMPs stated that they individualize treatments for their patients. TMP1 recommended a phase out plan of the allopathic medications as his drug kicked in. Six months was sufficient for a complete cure regardless of the patient. He also stated that “catching” the disease early would mean faster cure.

*“The patient must prepare the drug as instructed and take it three to four times a day. After this, the patient gets completely cured in six months. After six months, eat what you want but gauge yourself in the first one month.” (TMP 1)*

Herbalist 2 determined the duration of treatment based on how long the patient had had the disease and patient's weight. However, no weighing of patients was observed for dosage calculations. Stopping his drug before time caused the disease to return. *“If a patient has had the condition for four years, the treatment must go on for four years. Oil concoctions make the drug stronger so less of the drug is given.” (TMP 2)* Herbalist 3 advised that his drug be given only when the patient was unwell. When the patient improved, the drug was discontinued. All had doses for all patients which were arbitrary. Herbalist 3 and 4 mentioned that treatment durations and outcomes depended on the patient as all patients were different.

### Formulation of the herbal remedies

The TMPs had introduced certain excipients to improve on anti-diabetic products. These were grains, flavors and preservatives. This was done through trial and error. For instance, one herbalist had tried sodium benzoate as a preservative in diabetic treatments, disliked it and changed to natural preservatives; *Tamarindus indica* and *Eriobotrya japonica*. Honey and fenugreek were also used to improve taste of the herbal preparations.

### Adverse effects and the use of conventional medications

The TMPs reported that their drugs had minimal side effects compared to allopathic treatments. TMP 1 had patients report hypoglycemia while TMP 2 had reports of hyperglycemia. TMP 3 reported that sweating was the main side effect of his formulation. *“I have a secret formula to treat very high blood sugars in my patients. No matter how high the blood sugar rises, the patient cannot drop.” (TMP 1)*

The use of other herbs was the method of choice in treating these side effects. TMP 2 stated that he used *Hydenora abysinian* in small quantities to reduce side effects. Increasing the dose of his medication was also effective for hyperglycemia. TMP 3 said that there was no remedy for managing the excessive sweating. Change of drug and referring the patient for conventional medicines was done by all TMPs if adverse effects continued. None of the herbalists were opposed to use of conventional medication with their herbs. One herbalist said; *“If the patient is on conventional medicine, it will take only one month to wean them offthese drugs. If the patient is on insulin, the dose is halved until the patient completely abandons it. I however advise that the patient keeps the insulin and syringe nearby just in case.” (TMP 1)*

However, they all noted that although helpful, the conventional medications had many unpleasant effects. Herbs were considered safe by all TMPs as they were natural. One herbalist said that he had been using his formulation for a long time with no side effects. *“I have used this herbs for 10-15 years and it has no ill effects. It is a secret. 95% of the ones who knew of it have since died of old age.”(TMP 1)*

One herbalist commented: *“I add sodium bicarbonate to reduce toxicity of my drugs.”(TMP 2)* Three herbalist in this study allowed the researcher to observe their formulation processes and sample the drugs for analysis.

### Management of diabetic complications and claims of cure

All herbalists stated that their formulations reduced diabetic complications. However, three were more concerned with reduction of blood sugar levels as an objective marker. Only TMP1 was concerned with the healing of diabetic complications.

*“Sexual function in men, eye and kidney complications, memory, blood pressure all improve on my drug. A patient on my drug cannot not collapse due to high blood sugar. If a patient has an ulcer, it heals automatically. A close relative with sugar diseases who had swollen legs caused by hypertension got completely cured from my formulation. All diabetic complications improve.”(TMP 1)*

### Treatment monitoring and counseling

All herbalists monitored pre and post glucose levels using glucometers. Only herbalist 1 monitored improvement in diabetic complications. All TMPs keenly followed patient self-reported improvements of the signs and symptoms of T2DM. They were hesitant to talk of treatment failure but noted that patients who worsened were referred to conventional hospitals. Herbalist 1, 2 and 3 stated that treatment failure was caused by poor compliance and stress. One herbalist noted that stress contributes to treatment failure. *“A particular patient who had quarreled with her husband and was on my drug recorded very high blood sugars over of 30 mg/ml. She however did not drop. On counseling her, I advised that she must resolve the family issue. On sorting out the issue, her blood sugar dropped.”(TMP1)*

### Non-pharmacological interventions recommended by traditional medicine practitioners

Dietary advice was given by all TMPs. *“Dietary advice that I give must be followed. No sugar, starch, chapati and a controlled lifestyle with exercise.” (TMP 1)*

*“Avoid sugary meals. If taking meat, eat plants with an equal amount of protein for example finger millet, unpeeled potatoes and soya. Do physical exercises to increase sugar use by the body and to avoid liver and pancreatic problems.”(TMP 2)* All herbalist recommended lifestyle changes to their patients. All recommended that their patients should reduce sugar and salt intake. Traditional Kenyan diets and exercise was also recommended. It was noted that any patient not returning for treatment was considered cured. No efforts were made to trace patients.

### Cost of anti-diabetic herbal drugs

The average cost for the herbal anti-diabetic formulations was Ksh. 5000 per month without additional laboratory tests. Compared to conventional treatment, a monthly dose of 500 mg twice daily of metformin cost Ksh. 120 and that of glibenclamide once was daily Ksh. 60. TMP1 stated that depending on how you presented yourself, he would fix the cost so that all patients even the poor could benefit from his treatment. Payment on the spot was encouraged by all TMPs.

### Traditional medicine practitioners concerns about their practice

The TMPs interviewed in this study were keen to find out if their drugs were really effective. It was however noted that they wanted to maintain secrecy about their formulas. They were interested in registering their products but all had a low opinion about research institutions in Kenya whom they accused of stealing their formulations. All were concerned about the bad reputation that qualified herbalists had gotten from quacks. *“I would like to open a clinic at UoN so that my patients can be observed as I treat them. There is a similar project in Tanzania. I am confident that my drugs work but quacks have ruined the name of herbalist.”(TMP1)*

## Discussion

In concurrence with many studies done in Africa, all informants in this study were male [1, 5, 14, 15]. All were literate and used diverse treatments to manage T2DM. These modalities included; plant medicines, animal products, minerals, lifestyle advice and some modern conventional methods. Two TMPs stated family as a source of their knowledge. This is with agreement with other studies where family based apprenticeship was found to be the main source of knowledge of most TMPs [1]. Like noted by Ogbera and co-workers (2010), herbalists in this study were secretive about anti-diabetic management and formulations. They cited protection of family legacy and fear of loss of business as reasons for this [16].

Two TMPs practiced as full time herbalists. This implies that one can derive a livelihood from this trade. However, contrary to popular belief, this study found out that the cost of herbal remedies offered by TMPs were prohibitively expensive. This is in contradiction with the widely held belief that herbal drugs are affordable [2, 4, 17]. In contrast, Western allopathic medications are often considered more costly [3, 18]. The average cost for anti-diabetic Kenyan formulations in this study was Ksh. 5000 (55 USD) per month which was considerably higher than modern allopathic treatment. The herbalists in this study are based in cities and towns and it is a generally accepted fact that charges for services are ten times or more compared to those in the rural area. In comparison, the initial session for traditional anti-diabetic Chinese medicine was 75- 150 $ and follow-up visits were 65-100$ each. However, some insurers in China cover some costs [19]. All these costs by Kenyan standards of living, were found be high. In addition, traditional herbal medicine in Kenya is not covered by insurers and patients make out of pocket payments.

The causes of T2DM according to the herbalist were varied. One herbalist's assertion that it was caused by worms may have been his interpretation of literature. Chinese medicine believes that invisible worms called Gu are potential disease causing mechanisms of diabetes [20]. The herbalist's view was that he treated the “root cause” of the disease and hence cured the diabetes. Detoxifying and deworming was performed by this specific herbalist to treat his perceived root cause of T2DM. This idea is expressed by some Chinese patients who prefer traditional medicine because it treats the underlying cause of the disease [21].

Most patients were referrals from successfully treated patients. This was in agreement with other similar studies [4]. Advertising was however practiced by two TMPs. Records kept by two TMPs were provided and consisted of cards and books with demographic data. Patient follow up was none existent.

Diagnosis was symptom based with no physical exams witnessed. It is possible that physical exams were avoided due to lack of skill or equipment. In traditional Chinese medicine, which is a parallel traditional form of medicine, patient signs and symptoms such as odor of breath, pulse and palpation are done for chronic disease including diabetes. Detailed multi-system histories, including emotional and psychological responses are also done [19]. The use of hospital laboratory results was recorded with two TMPs who used this to aid in diagnosis and monitoring of T2DM. This could be attributed to the fact that these specific TMPs were better trained on management of the disease. The use of glucometers was positive although accuracy and correct usage was not ascertained [22]. The use of the glucometer by one TMP only if the patient did not know they had diabetes could be cost related since price and availability of strips was a concern by all TMPs.

The medicines ranged from herbs, to animal products and minerals. Taste and odor was noted as important characteristic when selecting herbs used in treatment of T2DM. One herbalist asserted that bitter herbs were best while the other stated that sweet tasting plants with pleasant odors were effective candidates. Similarly, in Nigeria, *Vernonia amygdalina*, a bitter tasting herb was cited by patients in the treatment diabetes. Its bitterness was thought to neutralize the sweetness in blood [16]. The view of one herbalist in this study was that anti-malarial herbs were a basis in his selection of anti-diabetic herbs. Indeed quinine and artemisinin are plant derive anti-malarial drugs with hypoglycemic effects [23, 24]. Minerals were included by one TMP since he perceived that lack of minerals was a cause of T2DM. This perception is in agreement with physiological studies. Minerals are thought to be deficient in diabetic patients and this worsens insulin deficiency [25]. Vanadium, chromium and magnesium have been reported to improve outcomes of diabetes [26]. The use of animal products by herbalists to treat T2DM could not be found in literature.

All TMPs used multi-component formulations which patients took as decoctions. In traditional Chinese medicine, it is thought that “formulations promote effective use of herbs” [19]. Chinese TMPs, divide their ingredients into four ingredients; principle, associate, adjutant and the guide ingredients. This organization is probably because Chinese traditional medicine is well researched and accepted compared to Kenyan herbal medicine. The Kenyan herbalist were observed to be highly secretive so that any information that could assist them decipher their ingredients was met with suspicion. A degree of agreement between the herbs used in managing T2DM could not be done in this study due to the highly competitive nature of the herbalists. The anti-diabetic formulations used by the patients were decoctions. Decoctions are known to have poor bioavailability [2]. The researchers observed that they were cumbersome to prepare and the measurements given to patients were not standardized.

All TMPs were aware on the importance of diet and exercise and used it as a treatment modality. Lifestyle changes reduce progression of T2DM and are seen to be as effective as drugs [2, 27].

The herbalists thought their products to be free from side effects since they were natural. Indeed, side effects are a key problem associated with allopathic medicines. Adverse effects were thought by the TMPs to be rare and patient specific [2]. One herbalist stated that excessive sweating was a side effect of his drug. This is a sign of hypoglycemia. Herbs are generally considered to be relatively safe with fewer adverse effects because they are from natural sources [18, 28]. This is a misconception since some herbs are known to be toxic [29]. In addition, they may be adulterated with toxic metals and microbes during preparation. Unscrupulous herbalists may also include conventional medications in herbal preparations to boost the performance of their herbs [30]. These dishonest herbalist were referred to as quacks by the TMPs and were a big concern to them.

Doses were observed to be arbitrary in this study regardless of weight or other conditions. Durations were individualized by the study herbalists. Early treatment was favored since “catching” the disease early would mean faster and easier cure. This view was also expressed by TMPs in a study in Uganda [4]. In Chinese traditional medicine, prescriptions are based on the patient's predominant symptoms. About sixteen sessions are sufficient in managing diabetes with follow-ups every 2-6 months. Traditional Chinese medicine is not however considered curative but rather it optimizes body function [19]. TMPs in this study considered that they drugs were curative. Monitoring was done by patients self-reporting and sometimes even over the phone. Self-report is always seen as undependable but is the most practical and cost effective method of self-care [31].

Several excipients were recorded which served various functions in the herbal medications. Addition of these substances was based on research and experimentation by the herbalists. Sodium bicarbonate was added to reduce toxicity of herbal formulations by one herbalist. It is thought that addition of sodium bicarbonate to the diet of patients with metabolic acidosis and chronic kidney disease is an affordable method that could halt kidney decline [32]. Preservatives and taste enhancers were also added. The safety of sodium benzoate as a preservative in food is established [33]. *Tamarindus indica* as a preservative has been found to be effective against all bacteria known to cause food spoilage but was ineffective against fungi [34]. No literature was found about the use of loquat as a preservative. However, loquat has been shown to produce tormentic acid that not only improves symptoms of diabetes but also increases insulin production [35]. It also contains polysaccharides that also increase insulin production^36^. *Tamarindus indica* has been used in India for treatment of diabetes and has been shown to have potent anti-diabetic action in animal studies [37]. Grains such as sorghum, millet and soya were added as carriers by one TMP but are known to have promising anti-diabetic effects [38]. Although honey and fenugreek (*Trigonella foenum - graecum*) were taste enhancers, they also have additional benefits in management of T2DM. Anti-diabetic medications that are combined with honey not only improve glycemic control, but they reduce oxidative damage [39]. Fenugreek acutely reduces postprandial glucose levels [40]. Canola oil and olive oil were added as diluents by one TMP to his anti-diabetic formulations. Canola oil is high in monounsaturates and has been shown to substantially reduce total cholesterol and low density lipoprotein. It also improves insulin sensitivity [41]. Olive oil is a free radical scavenger [42]. The addition of excipients may have unwittingly improved the anti-diabetic properties of the herbalist's drugs.

Drug interactions between these excipients and the polyherbal formulations are possible but were not investigated. Patients combining herbal drugs with allopathic herbs were also exposed to drug-drug interactions since the herbalists did not object to the combination. Drug interactions between conventional medicines and herbal formulations are known to occur [43]. There has been no local research on the use of these anti-diabetic herbal drugs with conventional medications.

## Conclusion

Knowledge and practice of the TMPs has a lot in common with allopathic medicine in terms of diagnosis, non-pharmacological management and risk of hypoglycemic reactions. Kenyan herbalists investigated in this study have incorporated some techniques and equipment used by allopathic practitioners.
